# Evaluating the Prognostic Accuracy of New Scores for In‐Hospital Outcomes in Cirrhotic Patients With Esophageal Variceal Bleeding

**DOI:** 10.1155/cjgh/1577589

**Published:** 2026-04-13

**Authors:** Khoa Phuoc Nguyen, Xung Van Nguyen, Nhan Duc Le, Trung Hieu Doan

**Affiliations:** ^1^ Department of Gastroenterology and Hepatology, Da Nang Hospital, Da Nang, Vietnam; ^2^ Intensive Care Unit, Da Nang Hospital, Da Nang, Vietnam; ^3^ Department of Geriatrics, Da Nang Hospital, Da Nang, Vietnam

**Keywords:** ABC score, cirrhosis, esophageal variceal bleeding, prognostic accuracy

## Abstract

**Background:**

Esophageal variceal bleeding (EVB) is a serious complication of cirrhosis and a major cause of upper gastrointestinal hemorrhage, carrying substantial risks of mortality and treatment failure. Prognostic scores are essential for guiding management. This study evaluated and compared the predictive accuracy of the ABC and MAP(ASH) scores with established models in cirrhotic patients with EVB.

**Methods:**

We retrospectively analyzed 278 cirrhotic patients admitted for EVB at Da Nang Hospital, Vietnam, between January 2022 and January 2025 who underwent endoscopic variceal ligation. Data were collected for ABC, MAP(ASH), AIMS65, and Glasgow−Blatchford scores. Primary outcomes were in‐hospital mortality and 5‐day treatment failure. Predictive performance was assessed using AUROCs and statistical comparisons.

**Results:**

The ABC score achieved the highest AUROC for predicting in‐hospital mortality (0.88), significantly surpassing the MAP(ASH), GBS, and AIMS65 scores (*p* < 0.001 for all pairwise comparisons). A similar trend was observed for predicting 5‐day treatment failure, where the ABC score again demonstrated the highest AUROC (0.79), outperforming both the GBS and AIMS65 scores; however, it showed comparable performance to MAP(ASH) (*p* = 0.19). In addition, the ABC score’s risk stratification (low, medium, and high) accurately differentiated patients with varying mortality and treatment failure rates.

**Conclusion:**

The ABC score is a highly effective and reliable tool for predicting in‐hospital mortality and early treatment failure in cirrhotic patients with EVB. While the MAP(ASH) score remains valuable for predicting early treatment failure, the ABC score offers superior overall prognostic accuracy. These findings suggest that the ABC score can guide clinical decisions, particularly in resource‐limited settings.

## 1. Introduction

Upper gastrointestinal (GI) bleeding represents a common and life‐threatening emergency with potentially severe outcomes [1]. In individuals with cirrhosis, the predominant etiology is bleeding from ruptured esophageal varices, which develop as a complication of portal hypertension. This hypertension results from impaired blood flow through the portal vein due to architectural distortion of the cirrhotic liver, causing increased venous pressure, progressive variceal dilation, and eventual rupture [2]. Current management strategies for esophageal variceal bleeding (EVB) focus on initial resuscitation to stabilize hemodynamics, administering vasoconstrictors (such as octreotide, somatostatin, or terlipressin), using prophylactic antibiotics, and performing urgent endoscopic interventions [3]. Although considerable progress has been made in managing cirrhosis and its associated complications, EVB still occurs frequently, occurring in 20%–40% of patients, with 6‐week mortality reported at approximately 10%–20% [4, 5]. This highlights the importance of developing accurate prognostic scoring systems to assess clinical outcomes in patients with variceal bleeding. These scoring systems allow clinicians to stratify patients according to the severity of their condition, enabling more personalized treatment strategies and timely interventions. By accurately identifying high‐risk individuals, these systems may help optimize patient management and significantly improve survival rates.

Currently, a variety of prognostic scoring systems have been proposed to assess outcomes in cirrhotic patients with variceal bleeding. Traditional scoring models such as Child−Pugh, Model for End‐Stage Liver Disease (MELD), Rockall, AIMS65, and Glasgow−Blatchford Bleeding Score (GBS) have proven useful in predicting these patients’ prognoses [6–9]. However, these systems have some restrictions, such as reliance on subjective factors and complex calculations, which can complicate their clinical application. In addition, emerging evidence also suggests that their accuracy in predicting outcomes such as rebleeding and mortality may not be as reliable as needed [10].

In 2019, Redondo‐Cerezo et al. introduced MAP(ASH), a straightforward preendoscopy score derived from a Spanish cohort and later confirmed in a global population [11]. This score, easy to remember, uses the acronym MAP(ASH) for mental status, ASA score, heart rate, albumin, systolic blood pressure, and hemoglobin. Designed to swiftly identify patients at higher risk of death, this score demonstrated strong predictive accuracy for mortality and interventions in upper GI bleeding while offering the benefit of being simpler to calculate than other existing risk scores [11]. A year later, building on similar principles, Laursen et al. developed the Age, Blood tests, and Comorbidities (ABC) score, praised for its simplicity and precision in predicting 30‐day mortality in both upper and lower GI bleeding. The ABC score was shown to surpass previously established prognostic scores in predictive accuracy [12]. A recent cohort study from Vietnam also demonstrated the effectiveness of the ABC score in predicting rebleeding and in‐hospital mortality in cirrhotic patients with variceal bleeding [13]. This further reinforces the value of the ABC score not only in evaluating a patient’s immediate condition but also in forecasting potential severe complications, enabling clinicians to make more informed and timely treatment decisions.

While numerous studies have explored the effectiveness of prognostic scores for GI bleeding, including both variceal and nonvariceal types, few have focused specifically on cirrhotic patients with variceal bleeding. Most existing research has evaluated the performance of individual scoring systems [11, 13, 14], and there has yet to be a study directly comparing these tools, especially newer ones such as the ABC and MAP(ASH) scores. For example, a recent study by Ky et al. [13] demonstrated that the ABC score is strongly associated with early re‐hemorrhage and in‐hospital mortality in cirrhotic patients with variceal bleeding. However, their study evaluated the ABC score in isolation and did not compare it to other widely used scoring systems such as MAP(ASH), GBS, and AIMS65. Moreover, their sample size was relatively small, and the authors themselves pointed out this limitation, suggesting that future research should compare the ABC score with other systems to determine the optimal prognostic tool. In light of this gap, we designed this retrospective study to compare the discriminatory power of the ABC and MAP(ASH) scores with other well‐known prognostic systems (GBS and AIMS65) in predicting in‐hospital mortality and 5‐day treatment failure in cirrhotic patients with EVB. Our study is novel in that it provides a thorough comparison of multiple scoring systems, helping to clarify which one is most effective at predicting both in‐hospital mortality and early treatment failure.

## 2. Methods

### 2.1. Study Design and Patient Cohort

This single‐center retrospective study encompasses all cirrhosis patients admitted to Da Nang Hospital, Vietnam, due to acute EVB and who underwent endoscopy band ligation from January 2022 to January 2025.

Patients were eligible for inclusion based on the following criteria: (1) age ≥ 18 years; (2) a confirmed diagnosis of cirrhosis, either from a documented medical history or determined through a combination of clinical presentation, laboratory findings, and imaging results; and (3) presentation with upper GI bleeding, manifesting as hematemesis, melena, or hematochezia, confirmed to originate from esophageal varices on endoscopy, followed by treatment with ligation.

Patients were excluded if they met any of the following conditions: (1) did not undergo endoscopy or had contraindications for the procedure; (2) presented with bleeding caused by alternative upper GI sources (e.g., peptic ulcers, gastritis, and portal hypertensive gastropathy) or from gastric varices, since our facility lacked Histoacryl injection capability during the study period; and (3) had already received endoscopic intervention at a different hospital within 7 days before hospitalization.

### 2.2. Patient Management

Since admission to the emergency department, emergency physicians promptly consulted the on‐call gastroenterologist to consider the need for endoscopy intervention. Endoscopy was generally performed after the patient had undergone initial resuscitation and their hemodynamic status had been stabilized. The decision to proceed, along with the proper timing for the procedure, was carefully determined based on the clinical judgment of the gastroenterologist. Once deemed appropriate, endoscopic variceal ligation (EVL) was carried out by a skilled endoscopist with expertise in managing such cases.

Regarding management, all patients were managed in accordance with the latest European Society of GI Endoscopy (ESGE) and Baveno VII recommendations [15, 16]. The initial approach focused on stabilizing the patient through fluid resuscitation and blood transfusions to ensure adequate tissue perfusion. To prevent infection, prophylactic antibiotics were administered, and vasoactive therapy was initiated. Octreotide was given as a 50 μg intravenous bolus, followed by a continuous infusion at a rate of 50 μg/hour for a duration of 2–5 days, with the infusion typically discontinued 48 h after achieving stable hemostasis. In high‐risk cases, such as severe initial bleeding or persistent hemoglobin decline despite successful EVL, the infusion was extended up to 5 days. Blood transfusions were provided when hemoglobin levels fell to ≤ 70 g/L to maintain levels between 7 and 9 g/dL.

### 2.3. Data Collection

To identify cirrhotic patients presenting with EVB, we systematically reviewed the electronic inpatient database of Da Nang Hospital throughout the study period. Patient eligibility was confirmed based on a thorough evaluation that included past medical history, clinical features, laboratory results, and findings from endoscopic examinations performed during admission. For individuals with multiple admissions due to variceal bleeding within the study timeframe, only the first hospitalization was considered in the analysis to maintain consistency.

A wide range of patient data was collected, including demographic details, medical history, underlying comorbidities, and key laboratory parameters at the time of admission, to calculate Child−Pugh, MELD, ABC, MAPASH, AIMS65, and Glasgow−Blatchford scores.

Clinical outcomes included in‐hospital mortality and 5‐day treatment failure. In‐hospital mortality was defined as death occurring during the same admission due to the bleeding episode or decompensated liver disease. Treatment failure within 5 days was defined, according to Baveno VII criteria, as either uncontrolled bleeding or rebleeding within the first 5 days [15].

### 2.4. Statistical Analysis

Qualitative variables were expressed as numbers and percentages (%), while quantitative variables were expressed as medians with interquartile ranges (IQRs).

The chi‐square test or Fisher’s exact test was applied to compare qualitative variables.

To evaluate the prognostic performance of the ABC, MAP(ASH), AIMS65, and Glasgow−Blatchford scoring systems, receiver operating characteristic (ROC) curve analyses were carried out. The area under the ROC curves (AUROCs) was calculated along with their 95% confidence intervals. The optimal cutoff point for each score was determined using the Youden index [17]. At this cutoff, sensitivity, specificity, positive predictive value (PPV), and negative predictive value (NPV) were calculated. AUROCs were compared using the method described by DeLong et al. [18].

Statistical significance was defined as a two‐sided *p* value below 0.05. All analyses were conducted with SPSS software (Version 29.0).

## 3. Results

### 3.1. Patient Characteristics

Overall, a total of 434 patients with cirrhosis were admitted to our hospital due to upper GI bleeding. After excluding 156 patients (52 who did not undergo endoscopy, 68 with nonvariceal bleeding, and 36 with gastric variceal bleeding), 278 eligible patients were enrolled in this study. The overall baseline characteristics of participants are presented in Table 1. The median age observed was 56 years (IQR: 50–64), with a notable male predominance, accounting for 86.7% of the cases, resulting in a male‐to‐female ratio of 6.5:1. Among the patients, 32.4% (90) had a prior history of variceal bleeding. The leading cause of cirrhosis was alcohol abuse, affecting 52.9% of patients, followed by chronic hepatitis B infection at 19.4%. Liver cancer was present in 16.2% (45) of the cases. Most patients (79.1%) were hospitalized primarily due to hematemesis. The majority of patients were classified in Child−Pugh Class B, accounting for 62.9%, followed by 23.7% in Class C and 13.3% in Class A. In addition, the majority of admitted patients had MELD scores exceeding 10, with a prevalence of 79.9%.

**TABLE 1:** Baseline characteristics of study participants at admission.

Patients features	Median (IQR) or *n* (%)
Age (years)	56 (50–64)
Gender: Male	241 (86.7)
Etiology	
HBV	54 (19.4)
HCV	19 (6.8)
Alcohol	147 (52.9)
HBV + alcohol	21 (7.6)
HCV + alcohol	1 (0.4)
Others	36 (12.9)
Symptoms at admission	
Hematemesis	220 (79.1)
Melena	196 (70.5)
Hematochezia	26 (9.4)
Child Pugh	
A	37 (13.3)
B	175 (62.9)
C	66 (23.7)
MELD score	13 (10–16)
History of bleeding	90 (32.4)
Liver cancer	45 (16.2)
Disseminated malignancy	17 (6.1)
Heart rate (beats per minute),	92.5 (85–103)
Systolic blood pressure (mmHg)	110 (100–120)
Hemoglobin (g/l	84 (68–103)
Platelet (10^9^/L)	98 (68–139)
AST (U/L)	73 (46–132)
ALT (U/L)	34 (24–52)
Bilirubin TP (μmol/L)	35 (21–56)
Albumin (g/L)	28 (24–31)
Urea (mmol/L)	6.8 (4.9–9.8)
Creatinine (μmol/L)	71 (60–87)
INR	1.4 (1.2–1.6)
ABC	5 (5–7)
MAPASH	3 (3–5)
GBS	10 (8–12)
AIMS65	1 (1–2)
Outcome:	
5‐day treatment failure	29 (10.4)
In‐hospital mortality	27 (9.7)

### 3.2. Clinical Outcomes

Among a total of 278 patients with GI bleeding due to ruptured esophageal varices, 27 patients (9.7%) died during hospital treatment, despite the successful application of EVL. Of these, 10 patients (37%) succumbed to severe liver disease, while 17 patients (63%) died due to severe rebleeding. Furthermore, our data found a 5‐day treatment failure rate of 10.4%, with 29 patients experiencing either failure to control bleeding or recurrent bleeding within this critical period (Table 1).

### 3.3. Death During Hospitalization

**FIGURE 1:**
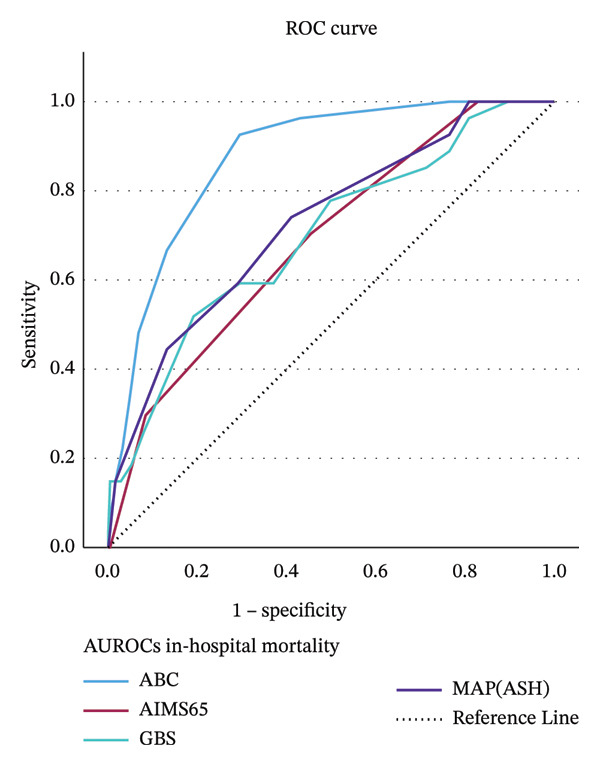
AUROCs of 4 different scores for forecasting in‐hospital death.

AUROCs of 4 different scores for predicting in‐hospital mortality are presented in Figure 1. The ABC score achieved the highest AUROC of 0.88 (95% CI: 0.82–0.93; *p* < 0.001), outperforming the MAP(ASH) with an AUROC of 0.73 (95% CI: 0.63–0.84; *p* < 0.001), the GBS with an AUROC of 0.70 (95% CI: 0.69–0.81; *p* < 0.001), and the AIMS65 score, which had an AUROC of 0.69 (95% CI: 0.59–0.79; *p* < 0.001) (Table 2). The AUROC of the ABC score was significantly superior to the other scoring systems, with *p* < 0.05 for all pairwise comparisons. While the AUROC of MAP(ASH) was notably higher than both GBS and AIMS65 (*p* < 0.001), there was no significant difference between GBS and AIMS65 (*p* = 0.84) (Table 3).

**TABLE 2:** Discriminative ability of the 4 scoring systems for in‐hospital death.

Score	AUROC	Cutpoint	Sensitivity (%)	Specificity (%)	PPV (%)	NPV (%)	*p* value
ABC	0.88 CI 0.82–0.93	7	79.7	78.7	28.7	97.3	< 0.001
MAP (ASH)	0.73 CI 0.63–0.83	4	66.7	65.0	17	94.8	< 0.001
GBS	0.70 CI 0.69–0.81	13	38.9	86.5	23.6	92.4	< 0.001
AIMS65	0.69 CI 0.59–0.79	2	50.0	73.1	16.6	93.2	< 0.001

**TABLE 3:** *p* values for pairwise comparisons among different scores.

In‐hospital death				5‐Day treatment failure		
MAP (ASH)	GBS	AIMS65	Score	AIMS65	GBS	MAP (ASH)
0.003	< 0.001	< 0.001	ABC	< 0.001	0.007	0.19
	< 0.001	< 0.001	MAP (ASH)	0.002	0.07	
		0.84	GBS	0.59		

The best cutoff values for the scoring systems were as follows: 7 for ABC, 4 for MAP(ASH), 13 for GBS, and 2 for AIMS65. Table 2 illustrates the sensitivity, specificity, PPV, and NPV for each score in relation to in‐hospital mortality.

### 3.4. 5‐Day Treatment Failure

**FIGURE 2:**
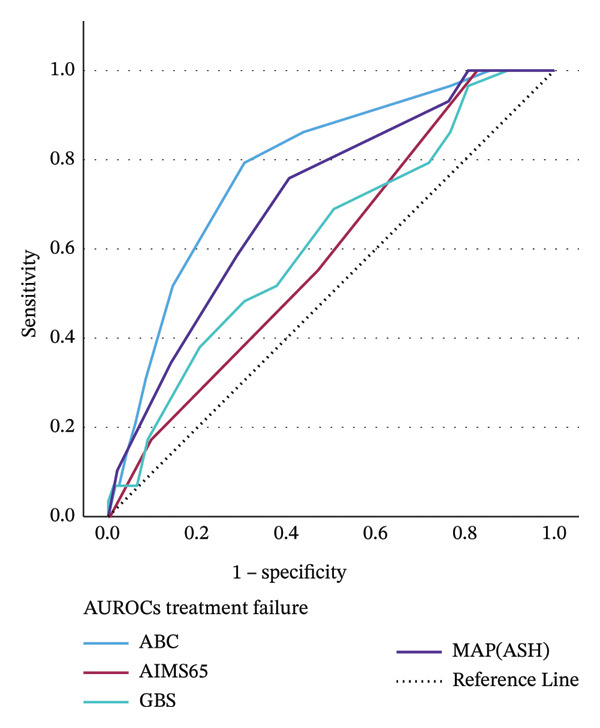
AUROCs of the 4 different scores for forecasting 5‐day treatment failure.

AUROCs of 4 different scores for predicting 5‐day treatment failure are presented in Figure 2. The AUROCs of 4 scores were as follows: ABC 0.79 (95% CI: 0.70–0.87; *p* < 0.001), MAP(ASH) 0.71 (95% CI: 0.62–0.81; *p* < 0.001), GBS 0.62 (95% CI: 0.52–0.73; *p* = 0.023), and AIMS65 0.59 (95% CI: 0.49–0.63; *p* = 0.07) (Table 4). The ABC score demonstrated better discriminative ability compared to the GBS and AIMS65 scores (*p* < 0.001), but no considerable difference was observed between ABC and MAP(ASH) scores (*p* = 0.19). The AUROC for MAP(ASH) was significantly greater than that of AIMS65 (*p* = 0.002). However, there were no significant differences between MAP(ASH) and GBS (*p* = 0.07) or between GBS and AIMS65 (*p* = 0.59) (Table 3).

**TABLE 4:** Discriminative ability of the 4 scoring systems for 5‐day treatment failure.

Score	AUROC	Cutpoint	Sensitivity (%)	Specificity (%)	PPV (%)	NPV (%)	*p* value
ABC	0.79 CI 0.70–0.87	7	65.5	77.5	25.3	95.1	< 0.001
MAP (ASH)	0.71 CI 0.62–0.81	4	67.3	65.3	18.3	94.5	< 0.001
GBS	0.62 CI 0.52–0.73	10	50.4	55.8	13.7	92.4	0.023
AIMS65	0.59 CI 0.49–0.6	1	77.6	35.2	12.2	93.1	0.066

Cutoff scores for ABC were 7, MAP(ASH) 4, GBS 10, and AIMS65 1. Table 2 depicts sensitivity, specificity, PPV, and NPV for each score regarding treatment failure within 5 days (Table 4).

### 3.5. Subanalysis for ABC Score

The ABC score categorizes patients into low (≤ 3), medium (4–7), and high (≥ 8) risk groups [12, 19]. In our study, 36, 191, and 51 patients were classified as low, medium, and high risk based on their ABC scores, respectively. The rates of in‐hospital mortality as well as treatment failure were significantly different among the three risk groups (*p* < 0.001), which corresponded well with the severity of their risk assessments. These findings are presented in detail in Table 5.

**TABLE 5:** Risk stratification with the ABC score.

ABC classification	In‐hospital mortality			Treatment failure in 5 days		
	No (%)	Yes (%)	*p*	No (%)	Yes (%)	*p*
High risk (≥ 8) *n* = 51	33 (64.7)	18 (35.3)	< 0.001	36 (70.6)	15 (29.4)	< 0.001
Moderate risk (4–7) *n* = 191	182 (95.3)	9 (4.7)		177 (92.7)	14 (7.3)	
Low risk (≤ 3) *n* = 36	36 (100)	0		36 (100)	0	

## 4. Discussion

Variceal hemorrhage is responsible for around 70% of GI bleeding cases in individuals with portal hypertension [20]. According to research in the United States, it was reported that 34,895 patients were hospitalized each year due to EVB [21, 22]. Furthermore, re‐hemorrhage and mortality rates within the first month remain alarmingly high, at 25.7% and 15.2%, respectively, mainly due to difficulties in controlling bleeding during the initial days [23, 24]. These findings underscore the pressing need for more accurate prognostic scoring systems to improve patient outcomes. Given these challenges, our study aimed to evaluate the prognostic performance of ABC and MAP(ASH) scores, alongside two well‐known scoring systems (GBS and AIMS65), in predicting outcomes during hospitalization in cirrhotic patients with EVB.

Our findings suggested that the ABC score was highly effective in predicting both in‐hospital mortality and 5‐day treatment failure in cirrhotic patients with EVB. While the MAP(ASH) score was also valuable and comparable to ABC in predicting early treatment failure, it did not outperform the ABC score in predicting in‐hospital death. These findings are consistent with a recent study from Vietnam by Ky et al. [13], which showed that the ABC score is strongly associated with early re‐hemorrhage and in‐hospital mortality in cirrhotic patients with variceal bleeding. However, their study focused solely on the ABC score, without comparing it to other widely used scoring systems such as GBS and AIMS65. In contrast, our study not only confirms the effectiveness of the ABC score in predicting in‐hospital mortality and 5‐day treatment failure but also highlights that, while the MAP(ASH) score is useful for predicting early treatment failure, it does not surpass the ABC score in predicting in‐hospital death.

In our study, the ABC score demonstrated superior performance over the MAP(ASH), GBS, and AIMS65 scores in predicting in‐hospital mortality, as evidenced by its significantly higher AUROC with *p* < 0.05 in all pair comparisons. This finding aligns with the work of Laursen et al. [12], who highlighted the superior accuracy of the ABC score in predicting mortality in both upper and lower GI bleeding across various patient groups. Similarly, Mules et al. [25] identified the ABC score as the most accurate among several commonly used scoring systems, with an AUROC of 0.85 for mortality prediction, outperforming GBS (0.71), AIMS65 (0.70), and Rockall (0.75); however, their study did not include the MAP(ASH) score. A comparable finding was also observed in a single‐center study from Korea performed on patients with peptic ulcer bleeding [26]. In addition, a recent retrospective study from Spain further supported the good performance of the ABC score, reporting an AUROC of 0.8 for predicting in‐hospital mortality in patients with upper GI bleeding [27]. Nevertheless, unlike our findings, that study observed nearly equivalent performance between the ABC and MAP(ASH) scores (0.80 vs. 0.79), which may be attributable to differences in study populations, as it included both variceal and no‐variceal bleeding cases, whereas our cohort focused on cirrhotic patients with confirmed variceal bleeding.

The superior performance of the ABC score in our study may be explained by its inclusion of parameters that more specifically reflect the clinical and pathophysiological characteristics of cirrhotic patients. The score allocates 2 points for the presence of cirrhosis and 2 points for hypoalbuminemia, both of which are common and clinically significant in this population. In our cohort, the majority of patients were classified as ASA Class 3 or 4, reflecting substantial systemic compromise. Furthermore, hypoalbuminemia is a well‐known marker of liver dysfunction and poor prognosis in cirrhosis, while elevated creatinine levels, often seen in patients with advanced liver disease or acute kidney injury, further worsen outcomes. Elevated blood urea nitrogen (BUN), frequently associated with massive variceal hemorrhage, also contributes to the ABC score and strengthens its prognostic value in this setting. Collectively, these variables offer a disease‐specific perspective that enhances the score’s discriminatory ability in cirrhotic patients. By contrast, the MAP(ASH) score, though easy to apply and broadly applicable, relies on more general parameters that may be less sensitive to the nuanced severity and complications of cirrhosis, potentially limiting its predictive precision in this subgroup.

In addition, the ABC score also showed acceptable performance in predicting 5‐day treatment failure, surpassing both GBS and AIMS65. A similar pattern was noted in a study by Ky et al. [13], which also demonstrated that the ABC score was a robust predictor of early rebleeding in cirrhotic patients with variceal bleeding. While MAP (ASH) had comparable performance to the ABC score for this secondary endpoint, it did not exhibit the same level of superiority as it did for in‐hospital mortality. According to research on elderly patients with upper GI bleeding, Li et al. [28] also found a similar discriminative ability between ABC and MAP(ASH) scores in predicting the risk of rebleeding (AUROC: 0.72 vs 0.73, respectively). This suggests that while the ABC score remains a strong predictor of both in‐hospital mortality and early treatment failure, MAP(ASH) can still play a significant role in identifying high‐risk patients, especially for mortality outcomes.

Interestingly, despite the high overall accuracy of the ABC score in predicting in‐hospital outcomes, our findings revealed that its risk categories (low, medium, and high) provided a clear and meaningful stratification of patients, with significant differences in both mortality and treatment failure rates. In our cohort, the high‐risk group (ABC score ≥ 8) showed markedly higher rates of in‐hospital mortality (35.3%) and 5‐day treatment failure (29.4%) compared to the moderate‐risk (4–7) and low‐risk (≤ 3) groups. This observation aligns with the findings of previous studies [12–14], where stratification using the ABC score was also shown to reliably predict 30‐day mortality. The significantly higher mortality and treatment failure rates in the high‐risk group (ABC score ≥ 8) emphasize the clinical value of the ABC score, highlighting the need for more intensive interventions and closer monitoring for these patients. This supports the ABC score’s potential to guide clinical decision‐making, ensuring that high‐risk individuals receive appropriate care to improve their outcomes.

In contrast, the MAP(ASH) score, while useful, did not demonstrate clear superiority over the ABC score in our study. This may be because MAP(ASH) includes parameters that, though clinically relevant, might be less specific for predicting the outcome of variceal bleeding in cirrhotic patients compared to the more tailored variables of the ABC score. Our findings support the conclusion of Redondo‐Cerezo et al. [11], who suggested that MAP(ASH), despite its ease of use, may not be as precise in predicting mortality in cirrhotic patients compared to other scores.

Moreover, although there is some evidence indicating that GBS and AIMS65 are reasonably effective in prognostication of short‐term mortality and rebleeding for patients with upper GI bleeding (AUROCs > 0.70) [25, 26, 28], our study, which focuses specifically on cirrhotic patients with variceal bleeding, found their performance to be less favorable, particularly in predicting 5‐day treatment failure. This is consistent with the results of a study by Aluizio et al. [10], which found that the predictive ability of GBS and AIMS65 was limited in patients with cirrhosis and variceal bleeding. Although these models have been widely used for GI bleeding in general, our data suggest that they are not effective in predicting outcomes in cirrhotic patients with variceal bleeding, possibly due to their more generalized approach that does not fully account for the complexities associated with liver cirrhosis, as well as its complications.

### 4.1. Limitations

This study has several strengths, including its quite large cohort of patients with cirrhosis and EVB, as well as the use of modern scoring systems. However, we acknowledge several limitations of this study. Initially, as this was a retrospective study conducted at a single center, the findings may not be broadly generalizable. Therefore, further large‐scale, multicenter studies, particularly prospective studies, are needed to confirm these findings. Second, while we focused on short‐term outcomes (mortality and treatment failure), the long‐term prognostic value of these scores remains to be explored. Finally, patients with gastric variceal bleeding were not included because Histoacryl injection therapy was not available at our institution during the study period, which may limit comparability with studies that did include such cases. In addition, none of the patients underwent preemptive or rescue TIPS or liver transplantation, primarily due to the high cost of these procedures and their lack of availability at our hospital and across other facilities in Vietnam. This limited access to TIPS and liver transplantation may contribute to higher in‐hospital mortality rates compared to countries with more widespread use of these interventions. However, the absence of both TIPS and liver transplantation also removes any potential bias toward improved outcomes, ensuring a more accurate evaluation of the prognostic scores.

## 5. Conclusions

In conclusion, the ABC score emerges as a highly effective and reliable tool for predicting in‐hospital mortality and early treatment failure in cirrhotic patients with EVB. Its simplicity and predictive accuracy make it a promising option for clinicians in resource‐limited settings. MAP(ASH), though valuable, did not surpass the ABC score in this specific clinical context.

**Nomenclature**
GI: Gastrointestinal
EVB: Esophageal variceal bleeding
ABC: Age, Blood tests, and Comorbidities
GBS: Glasgow−Blatchford Bleeding Score
MELD: Model for End‐Stage Liver Disease
EVL: Endoscopic variceal ligation
ESGE: European Society of Gastrointestinal Endoscopy
IQR: Interquartile ranges
ROC: Receiver operating characteristic
AUROC: Area under the receiver operating characteristic curve
HBV: Hepatitis B virus
HCV: Hepatitis C virus
AST: Aspartate aminotransferase
ALT: Alanine transaminase
INR: International normalized ratio
PT: Prothrombin time
TIPS: Transjugular intrahepatic portosystemic shunt

## Author Contributions

Conception and design: Khoa Phuoc Nguyen and Trung Hieu Doan. Data collection: Khoa Phuoc Nguyen and Xung Van Nguyen. Analysis and interpretation of the data: Khoa Phuoc Nguyen, Trung Hieu Doan, and Nhan Duc Le. Drafting of the article: Khoa Phuoc Nguyen. Critical revision of the article for important intellectual content: Khoa Phuoc Nguyen and Trung Hieu Doan. Study supervision: Trung Hieu Doan and Nhan Duc Le.

## Funding

The authors have nothing to report.

## Ethics Statement

This retrospective study complied with the ethical standards of the Declaration of Helsinki. The protocol received approval from the Internal Medicine Council of Da Nang Hospital (Approval number: 2025.25.27). Due to the retrospective design and the use of de‐identified patient data, informed consent was waived.

## Consent

Please see the Ethics Statement.

## Conflicts of Interest

The authors declare no conflicts of interest.

## Data Availability

The data that support the findings of this study are available on request from the corresponding author. The data are not publicly available due to privacy or ethical restrictions.

## References

[1] Stanley A. J. and Laine L. , Management of Acute Upper Gastrointestinal Bleeding, BMJ. (2019) 364, 10.1136/bmj.l536, 2-s2.0-85063516211. 30910853

[2] Qi X. , Li Y. , Li B. et al., Timing of Endoscopy in Cirrhotic Patients with Acute Variceal Bleeding: Protocol of a Multicenter Randomized Controlled Trial, Therapeutic Advances in Gastroenterology. (2024) 17, 10.1177/17562848241295452. PMC11558738 39539489

[3] Garcia-Tsao G. , Abraldes J. G. , Berzigotti A. , and Bosch J. , Portal Hypertensive Bleeding in Cirrhosis: Risk Stratification, Diagnosis, and Management: 2016 Practice Guidance by the American Association for the Study of Liver Diseases, Hepatology. (2017) 65, no. 1, 310–335, 10.1002/hep.28906, 2-s2.0-85007173739. 27786365

[4] Garcia-Tsao G. and Bosch J. , Management of Varices and Variceal Hemorrhage in Cirrhosis, New England Journal of Medicine. (2010) 362, no. 9, 823–832, 10.1056/nejmra0901512, 2-s2.0-77649334176. 20200386

[5] de Franchis R. , Expanding Consensus in Portal Hypertension: Report of the Baveno VI Consensus Workshop: Stratifying Risk and Individualizing Care for Portal Hypertension, Journal of Hepatology. (2015) 63, no. 3, 743–752, 10.1016/j.jhep.2015.05.022, 2-s2.0-84939251317. 26047908

[6] Ryan K. , Malacova E. , Appleyard M. , Brown A. F. , Song L. , and Grimpen F. , Clinical Utility of the Glasgow Blatchford Score in Patients Presenting to the Emergency Department with Upper Gastrointestinal Bleeding: a Retrospective Cohort Study, Emergency Medicine Australasia. (2021) 33, no. 5, 817–825, 10.1111/1742-6723.13737. 33543572

[7] Altamirano J. , Zapata L. , Augustin S. et al., Predicting 6-week Mortality After Acute Variceal Bleeding: Role of Classification and Regression Tree Analysis, Annals of Hepatology. (2009) 8, no. 4, 308–315, 10.1016/s1665-2681(19)31743-0. 20009129

[8] Aluizio C. L. , Nagasako C. K. , Sampaio D. P. et al., Mo1150 Application of Rockall, Blatchford and Aims65 Scores to Risk Stratification for Acute Variceal Bleeding, Gastrointestinal Endoscopy. (2018) 87, no. 6, 10.1016/j.gie.2018.04.1911.

[9] Alia M. S. A. , Elsawy A. A. , Elarabawy R. A. , and Hegazy H. M. , Predictors of Early Rebleeding After Endoscopic Therapy of First Variceal Bleeding in Liver Cirrhosis, Egyptian Liver Journal. (2021) 11, no. 1, 10.1186/s43066-021-00119-2.

[10] Aluizio C. L. S. , Montes C. G. , Reis G. F. S. R. , and Nagasako C. K. , Risk Stratification in Acute Variceal Bleeding: far from an Ideal Score, Clinics. (2021) 76, 10.6061/clinics/2021/e2921. PMC8221560 34190855

[11] Redondo-Cerezo E. , Vadillo‐Calles F. , Stanley A. J et al., MAP(ASH): A New Scoring System for the Prediction of Intervention and Mortality in Upper Gastrointestinal Bleeding, Journal of Gastroenterology and Hepatology. (2020) 35, no. 1, 82–89, 10.1111/jgh.14811, 2-s2.0-85071232156. 31359521

[12] Laursen S. B. , Oakland K. , Laine L. et al., ABC Score: A New Risk Score that Accurately Predicts Mortality in Acute Upper and Lower Gastrointestinal Bleeding: an International Multicenter Study, Gut. (2021) 70, no. 4, 707–716, 10.1136/gutjnl-2019-320002. 32723845

[13] Ky T. D. , Trang N. T. H. , and Binh M. T. , Predictive Significance of the ABC Score for Early Re-Hemorrhage and In-Hospital Mortality in High-Risk Variceal Bleeding Among Cirrhotic Patients, Diagnostics. (2023) 13, no. 23, 10.3390/diagnostics13233570. PMC10706279 38066811

[14] Saade M. C. , Kerbage A. , Jabak S. , Makki M. , Barada K. , and Shaib Y. , Validation of the New ABC Score for Predicting 30-day Mortality in Gastrointestinal Bleeding, BMC Gastroenterology. (2022) 22, no. 1, 10.1186/s12876-022-02374-y. PMC9209314 35729498

[15] de Franchis R. , Bosch J. , Garcia-Tsao G. et al., Baveno VII-Renewing Consensus in Portal Hypertension, Journal of Hepatology. (2022) 76, no. 4, 959–974, 10.1016/j.jhep.2021.12.022. 35120736 PMC11090185

[16] Gralnek I. M. , Camus Duboc M. , Garcia-Pagan J. C. et al., Endoscopic Diagnosis and Management of Esophagogastric Variceal Hemorrhage: European Society of Gastrointestinal Endoscopy (ESGE) Guideline, Endoscopy. (2022) 54, no. 11, 1094–1120, 10.1055/a-1939-4887. 36174643

[17] Youden W. J. , Index for Rating Diagnostic Tests, Cancer. (1950) 3, no. 1, 32–35. 15405679 10.1002/1097-0142(1950)3:1<32::aid-cncr2820030106>3.0.co;2-3

[18] DeLong E. R. , DeLong D. M. , and Clarke-Pearson D. L. , Comparing the Areas Under Two or More Correlated Receiver Operating Characteristic Curves: A Nonparametric Approach, Biometrics. (1988) 44, no. 3, 837–845, 10.2307/2531595, 2-s2.0-0023710206. 3203132

[19] Tsai H. H. , Prognostic Risk Score for Gastrointestinal Bleeding: which One is Best?, GastroHep. (2021) 3, no. 1, 10.1002/ygh2.442.

[20] Guinazu C. , Fernández Muñoz A. , Maldonado M. D. et al., Assessing the Predictive Factors for Bleeding in Esophageal Variceal Disease: A Systematic Review, Cureus. (2023) 15, no. 11, 10.7759/cureus.48954. PMC10725706 38106778

[21] Siddiqui M. T. , Bilal M. , Haq K. F. , Nabors C. , Schorr-Lesnick B. , and Wolf D. C. , Seasonal Impacts on the Incidence of Esophageal Variceal Hemorrhage: A Nationwide Analysis Across a Decade, Clin Endosc. (2020) 53, no. 2, 189–195, 10.5946/ce.2019.094. 31878767 PMC7137566

[22] Seo Y. S. , Prevention and Management of Gastroesophageal Varices, Clinical and Molecular Hepatology. (2018) 24, no. 1, 20–42, 10.3350/cmh.2017.0064, 2-s2.0-85057246044. 29249128 PMC5875194

[23] Jairath V. , Rehal S. , Logan R. et al., Acute Variceal Haemorrhage in the United Kingdom: Patient Characteristics, Management and Outcomes in a Nationwide Audit, Digestive and Liver Disease. (2014) 46, no. 5, 419–426, 10.1016/j.dld.2013.12.010, 2-s2.0-84898870933. 24433997

[24] Thabut D. and Bernard-Chabert B. , Management of Acute Bleeding From Portal Hypertension, Best Practice & Research Clinical Gastroenterology. (2007) 21, no. 1, 19–29, 10.1016/j.bpg.2006.07.010, 2-s2.0-33846130915. 17223494

[25] Mules T. C. , Stedman C. , Ding S. et al., Comparison of Risk Scoring Systems in Hospitalised Patients who Develop Upper Gastrointestinal Bleeding, GastroHep. (2021) 3, no. 1, 5–11, 10.1002/ygh2.436.

[26] Sakong H. , Moon H. S. , Choi S. W. , Kang S. H. , Sung J. K. , and Jeong H. Y. , ABC Score is an Effective Predictor of Outcomes in Peptic Ulcer Bleeding, Medicine (Baltimore). (2022) 101, no. 49, 10.1097/md.0000000000031541. PMC9750577 36626500

[27] Jimenez-Rosales R. , Lopez-Tobaruela J. M. , Lopez-Vico M. , Ortega-Suazo E. J. , Martinez-Cara J. G. , and Redondo-Cerezo E. , Performance of the New ABC and MAP(ASH) Scores in the Prediction of Relevant Outcomes in Upper Gastrointestinal Bleeding, Journal of Clinical Medicine. (2023) 12, no. 3, 10.3390/jcm12031085. PMC9917936 36769733

[28] Li Y. , Lu Q. , Wu K. , and Ou X. , Evaluation of Six Preendoscopy Scoring Systems to Predict Outcomes for Older Adults with Upper Gastrointestinal Bleeding, Gastroenterology Research and Practice. (2022) 2022, 9334866–9334868, 10.1155/2022/9334866. 35136407 PMC8818397

